# Sodium Valproate Induces Cell Senescence in Human Hepatocarcinoma Cells

**DOI:** 10.3390/molecules181214935

**Published:** 2013-12-04

**Authors:** Hong-Mei An, Yong-Fei Xue, Yan-Li Shen, Qin Du, Bing Hu

**Affiliations:** 1 Department of Science and Technology, Longhua Hospital, Shanghai University of Traditional Chinese Medicine, Shanghai 202032, China; E-Mail: lhsoatp@163.com; 2 Department of Oncology, Center Hospital of Nanyang, Nanyang, Henan 473000, China; E-Mails: xyf820@sina.com (Y.-F.X.); shenyanli1981@126.com (Y.-L.S.); 3 Department of Oncology, Longhua Hospital, Shanghai University of Traditional Chinese Medicine, Shanghai 202032, China; E-Mail: lsduqin123@126.com; 4 Institute of Traditional Chinese Medicine in Oncology, Longhua Hospital, Shanghai University of Traditional Chinese Medicine, Shanghai 202032, China

**Keywords:** hepatocarcinoma, valproic acid sodium salt, cell senescence

## Abstract

Hepatocarcinogenesis is associated with epigenetic changes, including histone deacetylases (HDACs). Epigenetic modulation by HDAC inhibition is a potentially valuable approach for hepatocellular carcinoma treatment. In present study, we evaluated the anticancer effects of sodium valproate (SVP), a known HDAC inhibitor, in human hepatocarcinoma cells. The results showed SVP inhibited the proliferation of Bel-7402 cells in a dose-dependent manner. Low dose SVP treatment caused a large and flat morphology change, positive SA-β-gal staining, and G0/G1 phase cell cycle arrest in human hepatocarcinoma cells. Low dose SVP treatment also increased acetylation of histone H3 and H4 on p21 promoter, accompanied by up-regulation of p21 and down-regulation of RB phosphorylation. These observations suggested that a low dose of SVP could induce cell senescence in hepatocarcinoma cells, which might correlate with hyperacetylation of histone H3 and H4, up-regulation of p21, and inhibition of RB phosphorylation. Since the effective concentration inducing cell senescence in hepatocarcinoma cells is clinically available, whether a clinical dose of SVP could induce cell senescence in clinical hepatocarcinoma is worthy of further study.

## 1. Introduction

Hepatocellular carcinoma (HCC) is one of the most frequent malignancies worldwide [1]. The incidence of HCC is increasing due to hepatitis B and C virus infections [1,2]. Despite advances in diagnosis and treatment, HCC remains the third leading cause of cancer death worldwide [3,4]. During the past two decades, the incidence of HCC in the United States has tripled, while the overall 5-year survival rate has remained below 12% [5]. The high morbidity and mortality of HCC underscores the need to develop new therapeutic approaches for HCC treatment.

Accumulated evidence suggests that epigenetic changes, such as DNA methylation, histone modifications and RNA-mediated gene silencing, may contribute to hepatocarcinogenesis [6]. Hepatitis C virus infection may up-regulate protein phosphatase 2A, inhibit histone H4 methylation/acetylation and histone H2AX phosphorylation, which leads to significant changes to the expression of genes for hepatocarcinogenesis [7]. Hepatitis B virus X protein, a viral oncoprotein from hepatitis B virus, may induce histone deacetylase 1 (HDAC1) expression and enhance hypoxia signaling in hepatocellular carcinoma cells [8]. HDACs are overexpressed in HCC, which is associated with aggressiveness of HCC, and may be a useful biomarker for predicting the outcome of the patients with HCC [9,10]. Epigenetic modulation by HDACs inhibition is a potentially valuable approach for HCC therapy.

Valproic acid, a well-tolerated and widely used anti-convulsant, has been recognized as a HDAC inhibitor [11]. Valproic acid has displayed pro-apoptotic activities against various cancer cells, including prostate cancer [12], endometrial cancer [13], thyroid cancer [14], myeloma [15], and gastric cancer [16]. In addition to apoptosis, valproic acid or sodium valproate (SVP) have been confirmed to be effective in inducing cell senescence in medulloblastoma, head and neck cancer, and leukemia cells [17,18,19]. However, the effect of SVP on cell senescence in hepatocarcinoma cells remains unknown. In present study, we observed that, at clinical available doses, SVP induced cell senescence in hepatocarcinoma cells and accompanied by hyperacetylation of histone H3 and H4, up-regulation of p21 and down-regulation of RB phosphorylation.

## 2. Results and Discussion

### 2.1. SVP Inhibits the Proliferation of Bel-7402 Cells

**Figure 1:**
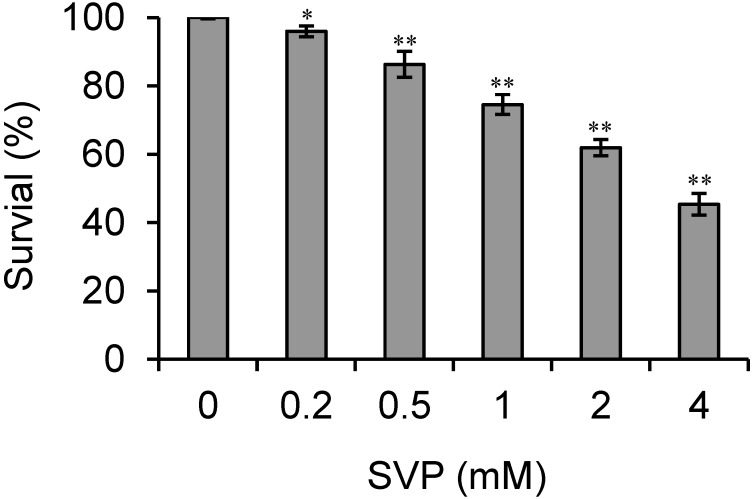
Effects of SVP on proliferation of Bel-7402 cells. Human hepatocarcinoma Bel-7402 cells were treated with different concentrations of SVP for 24 h, and cell viability was evaluated by CCK-8 assay. Data shown are representative of three independent experiments. * *p* < 0.05, *versus* control group. ** *p* < 0.01, *versus* control group.

Clinical concentrations of SVP range from 0.2 mM (the typical cerebrospinal fluid concentration) to 5.1 mM (associated with life-threatening toxicities, such as coma) [17], so we tested the effects of SVP on Bel-7402 cells proliferation at a final concentration of 0.2–4 mM. As shown in Figure 1, SVP inhibited the proliferation of Bel-7402 cells in a dose-dependent manner (*p* < 0.05).

### 2.2. SVP Activates SA-β-gal in Bel-7402 Cells

**Figure 2:**
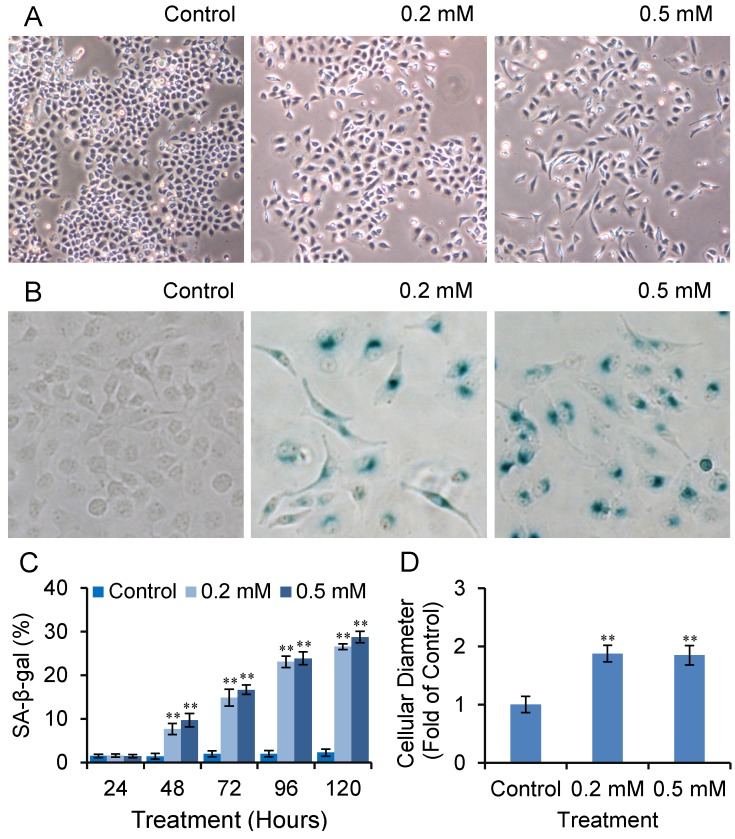
SVP activated SA-β-gal in Bel-7402 cells. Bel-7402 cells were treated with 0.2 and 0.5 mM SVP, and subjected to SA-β-gal staining. (**A**) Cell morphological change after SVP treatment (120 h, ×40). (**B**) Typical SA-β-gal staining of SVP treated (120 h) and untreated Bel-7402 cells (×100). (**C**) SA-β-gal-positive cells were counted and expressed as mean ± S.D. (**D**) Diameter of SA-β-gal positive cells were measured and expressed as fold of control negative cells. Data illustrated are representative of three independent experiments. ** *p* < 0.01, *versus* control group.

We observed that after long-term treatment with high doses of SVP, Bel-7402 cells were gradually dead and floated. At low doses of SVP treatment (0.2 mM and 0.5 mM, both of those doses are clinical available [17]), Bel-7402 cells were gradually exhibited a large and flattened morphology, reminiscent cell senescence (Figure 2A). We further performed senescence-associated β-galactosidase (SA-β-gal) staining. As shown in Figure 2B and C, SVP treatment resulted in SA-β-gal-positive staining which was started from 48 h and peaked on 120 h and compared with controls (*p* < 0.01). The cell diameter of SA- SA-β-gal-positive cells were wider than SA-β-gal-negative cells (*p* < 0.01).

### 2.3. SVP Arrests Bel-7402 Cells in G0/G1 Phase

**Figure 3:**
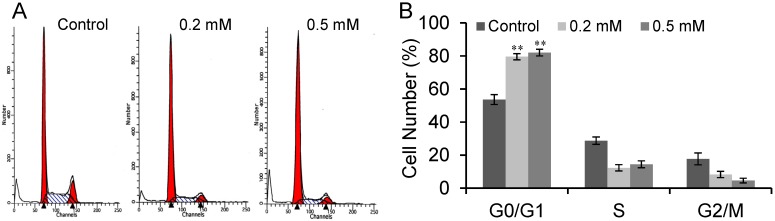
SVP induced cell cycle arrest in Bel-7402 cells. Bel-7402 cells were treated with 0.2 and 0.5 mM SVP for 120 h, and cell cycle distribution were detected by flow cytometric analysis (**A**), and expressed as mean ± S.D. (**B**) Data shown are representative of three independent experiments. ** *p* < 0.01, *versus* control group.

Cell senescence is a state of stable irreversible proliferation arrest that characterized by large and flattened morphology, elevated SA-β-gal activity and cell cycle arrest [20,21,22]. We also detected cell cycle distribution of SVP treated Bel-7402 cells. Flow cytometric analysis revealed that the cell cycle of SVP-treated Bel-7402 cells was arrested (*p* < 0.01) in the G0/G1 phase (Figure 3). These observations suggested that SVP may induce senescence in Bel-7402 cells.

### 2.4. Effects of SVP on the Expression of Senescence Associated Genes

**Figure 4:**
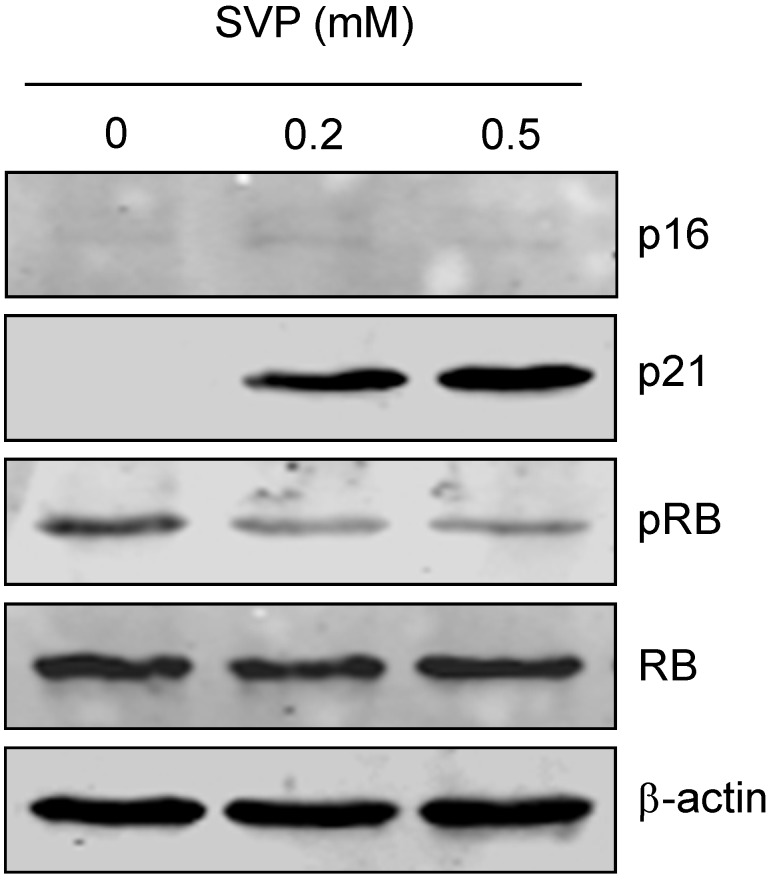
Effects of SVP on expression of cell senescence regulatory genes in Bel-7402. Bel-7402 cells were collected after 0.2 and 0.5 mM of SVP treatment for 120h, and subjected to western blots using antibody against p16, p21, RB and pRB. β-actin was used as a loading control.

The effects of SVP on the expression of senescence associated genes in Bel-7402 cells were detected by western blotting. As shown in Figure 4, treatment with low doses of SVP caused an up-regulation in the p21 expression, and down-regulation of RB phosphorylation. However, expression p16 and RB did not change after SVP treatment.

### 2.5. SVP Induces Hyperacetylation of Histone H3 and H4

**Figure 5:**
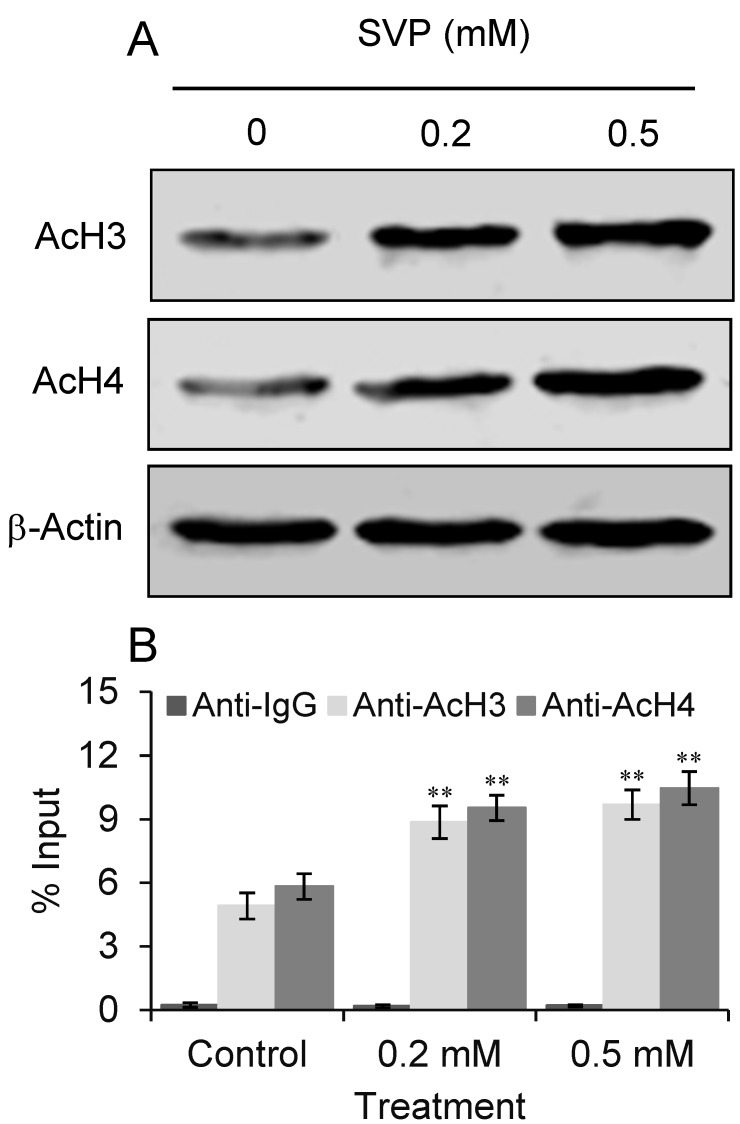
(**A**) SVP induced hyperacetylation of histone H3 and H4. Bel-7402 cells were collected after 0.2 and 0.5 mM of SVP treatment for 120 h, and subjected to western blotting using antibody against acetylated histone H3 (AcH3) and H4 (AcH4). β-actin was used as a loading control. (**B**) ChIP-qPCR analysis using AcH3 and AcH4 antibody or control IgG. Precipitated genomic DNA was amplified by qPCR and expressed as percentage of the total input genomic DNA. The result of three independent experiments was shown as mean ± S.D. ** *p* < 0.01, *versus* control group.

HDACs regulate gene transcription by deacetylating α-acetyl lysine that resides within the NH2-terminal tail of core histones, including histone H3 and H4. The effects of SVP on acetylation of histone H3 and H4 in Bel-7402 cells were detected by western blot. As shown in Figure 5A, histone H3 and H4 were hyperacetylated after low doses of SVP treatment. To ask if SVP induced p21 expression through increased histone H3 and H4 acetylation on p21 promoter, chromatin immunoprecipitation (ChIP) assays were performed by using acetylated histone H3 and H4 antibodies or control IgG. Furether qPCR revealed that significant increases of p21 promoter fragments were found in ChIP precipitated materials from SVP treated Bel-7402 cells (Figure 5B). These observations suggested increased acetylation of histone H3 and H4 on p21 promoter upon SVP treatment.

### 2.6. SVP Induces Cell Senescence in Bel-7404 Cells

**Figure 6:**
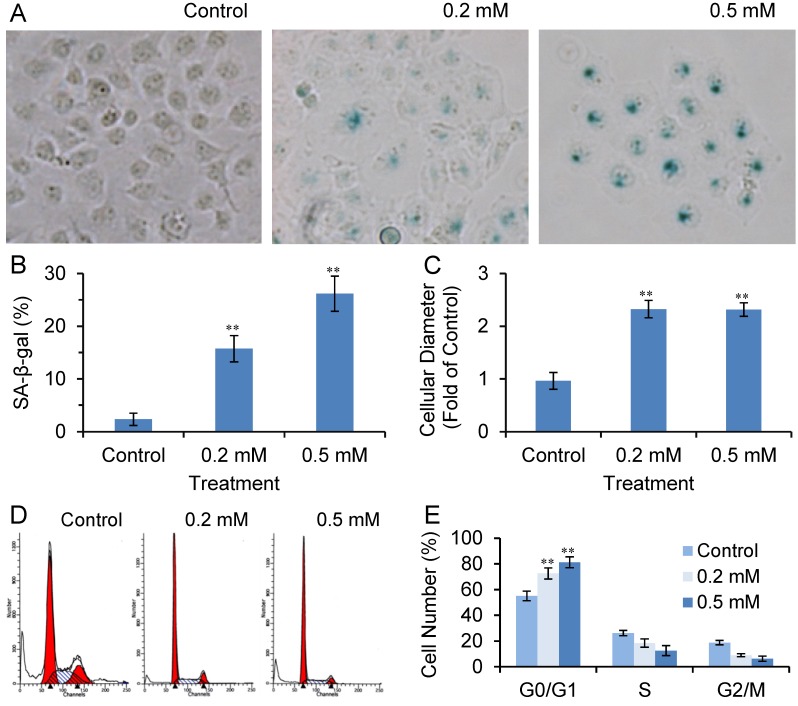
SVP induced cell senescence in Bel-7404 cells. Bel-7404 cells were treated with 0.2 and 0.5 mM SVP for 120 h, and subjected to SA-β-gal staining (**A**, ×100) and flow cytometric analysis (**D**). (**B**) SA-β-gal-positive cells were counted and expressed as mean ± S.D. (**C**) Diameter of SA-β-gal positive cells were measured and expressed as fold of control negative cells. (**E**) Cell cycle distribution are expressed as mean ± S.D. Data illustrated are representative of three independent experiments. ** *p* < 0.01, *versus* control group.

We also tested the effects of SVP in p53 mutated human hepatocarcinoma Bel-7404 cells. As shown in Figure 6, SVP treatment caused a large and flattened morphological change, SA-β-gal-positive staining and G0/G1 arrest in Bel-7404 cells. These observations suggested that SVP induced senescence in Bel-7404 cells.

### 2.7. SVP Induces p21 Expression and Inhibits RB Phosphorylation in Bel-7404 Cells

**Figure 7:**
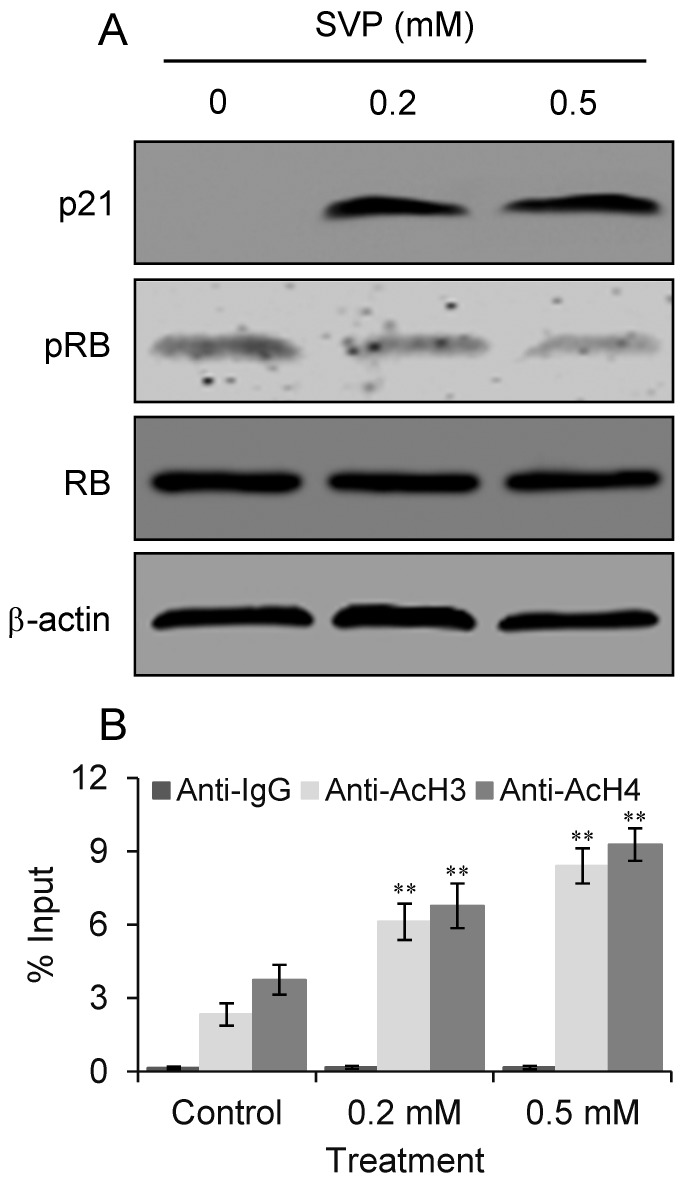
Effects of SVP on expression of cell senescence regulatory genes in Bel-7404 cells. (**A**) Bel-7404 cells were treated with 0.2 and 0.5 mM of SVP for 120h, and subjected to western blots using antibody against p21, pRB and RB. β-actin was used as a loading control. (**B**) ChIP-qPCR analysis using AcH3 and AcH4 antibody or control IgG. Precipitated genomic DNA was amplified by qPCR and expressed as percentage of the total input genomic DNA. The result of three independent experiments was shown as mean ± S.D. ** *p* < 0.01, *versus* control group.

Western blot was performed to detect p21 expression and RB phosphorylation in Bel-7404 cells. As shown in Figure 7A, treatment with low doses of SVP upregulated p21 expression and inhibited RB phosphorylation in Bel-7404 cells. The RB expression was not changed after SVP treatment. Further ChIP-qPCR assay revealed that increased acetylation of histone H3 and H4 on p21 promoter upon SVP treatment (Figure 7B).

### 2.8. Discussion

Cell senescence is a state of stable irreversible cell cycle arrest provoked by a variety of stimuli. Senescent cells maintain some metabolic activity, but can no longer proliferate, even stimulated with mitogens. Senescent cell is usually characterized by large and flattened morphology, elevated SA-β-gal activity and cell cycle arrest [20,21,22]. Cell senescence plays an important role in suppressing tumorigenesis, and has been identified as one of the mechanisms for anticancer therapy [21,22,23]. Chemotherapeutic agents such as cisplatin, doxorubicin, SN-38, and camptothecin have been reported to inhibit growth of cancer cells via cell senescence [23,24,25,26].

Accumulated studies demonstrated valproic acid or SVP are capable of inhibiting cancer cell growth through induction of apoptosis, cell cycle arrest or cell senescence [14,15,16,17,18,19]. However, the toxic and side effects have limited the potential use of SVP for cancer treatment. Since the clinical available concentration of SVP ranges from 0.2 mM (the typical cerebrospinal fluid concentration) to 0.6 mM (the typical therapeutic serum concentration). In present study, we observed that clinical available dose of SVP treatment caused a large and flat morphology change, positive SA-β-gal staining, and G0/G1 phase cell cycle arrest, which suggest clinical available dose of SVP treatment may induce cell senescence in hepatocarcinoma cells.

The occurrence of cell senescence is closely related to the activation of the p21/pRB/E2F or p16/pRB/E2F signaling pathway [27,28]. p21 also known as cyclin-dependent kinase inhibitor 1A (CDKN1A), CDK-interacting protein 1 (CIP1) or wildtype p53-activated fragment 1 (WAF1) [29,30]. p21 can inhibit a variety of cyclin/CDK complexes and induce the hypophosphorylation or dephosphorylation of protein RB. Hypophosphorylated pRB binds to E2F and prevents it from activating target genes that are essential in the cell cycle and may lead to cell cycle arrest. The present study showed p21 was upregulated by low dose of SVP treatment accompanied by downregulation of RB phosphorylation. These observations suggested SVP induced cell senescence may associated with up-regulation of p21 and downregulation of RB phosphorylation.

Transcription of p21 can be activated by p53 upon DNA damage or other stimuli. HDAC inhibitors activate the expression of p21 mainly in a p53-independent manner [30]. It has been reported HDACs inhibitors, such as statins, suberoylanilide hydroxamic acid or HDAC2 siRNA, may activate p21 expression through histones acetylation on its promoter [31,32,33]. In present study, we observed that SVP treatment upregulated p21 expression accompanied by increased acetylation of histone H3 and H4 on p21 promoter in both wild-type p53 (Bel-7402) and mutant p53 (Bel-7404) cells. These observations suggested SVP induced p21 expression in human hepatocarcinoma cells are irrelevant to p53 and may result from histones H3 and H4 acetylation.

## 3. Experimental

### 3.1. Chemicals and Reagents

Sodium valproate was purchased from Sigma-Aldrich (St. Louis, MO, USA). RPMI1640 medium and fetal bovine serum was obtained from Hyclone (Logan, UT, USA). Cell Counting Kit-8 (CCK-8) was from Dojindo (Kumamoto, Japan). Antibodies against acetylated histone H3 (AcH3) and H4 (AcH4), p16, p21, RB, pRB, and β-actin were from Cell Signaling Technology (Danvers, MA, USA). Senescence β-Gal Staining Kit was also purchased from Cell Signaling Technology. ChIP Assay Kit was obtained from Beyotime Institute of Biotechnology (Haimen, Jiangsu, China).

### 3.2. Cell Culture

Human hepatocarcinoma Bel-7402 and Bel-7404 cells were obtained from the Cell Bank of the Type Culture Collection of the Chinese Academy of Sciences (Shanghai, China). Hepatocarcinoma cells were grown in RPMI1640 medium with 10% FBS and 1% Pen-Strep, and maintained at a 37 °C in a humidified incubator with a 5% CO_2_ atmosphere.

### 3.3. Cell Proliferation Assay

Cells in logarithmic growth phase were seeded into 96-well plate (4 × 10^3^ cells/well) and allowed to attach for 24 h before treatment. The cells were exposed to various doses of SVP for 24 h, and cell viability was evaluated by using the CCK-8 colorimetric assay according to the manufacturer’s instructions. The cell survival rate was calculated as follows: cell survival rate (%) = experimental OD value/control OD value × 100%.

### 3.4. Senescence-Associated β-Galactosidase Staining

Hepatocarcinoma cells (3 × 10^4^) were plated in 35-mm-diameter plates and treated with different dose of SVP for 24 to 120 h. Senescence-associated β-galactosidase activity [20] was detected by Senescence β-Gal Staining Kit according to the manufacturer’s protocol, and observed under microscope.

### 3.5. Flow Cytometric Analysis

At the end of treatment, hepatocarcinoma cells were collected and washed with phosphate-buffered saline (PBS), fixed in 70% ethanol at 4 °C and treated with 10 mg/mL RNase for 30 min at 37 °C. Finally, cells were stained with PI (50 μg/mL) and analyzed in a FACScalibour flow cytometer (Becton Dickinson, Franklin Lakes, NJ, USA).

### 3.6. Western Blot

Western blot were performed as described previously [34,35]. Briefly, collected cells were lysed and subjected to 8%–12% SDS-PAGE gel, and transferred onto a nitrocellulose membrane (Amersham Biosciences, Buckinghamshire, UK). The transferred membrane were blocked with 5% non-fat milk, washed, and probed with the indicated antibodies. Blots were then washed and incubated with IRDye 700- and IRDye 800-conjugated secondary antibodies (Rockland Immunochemicals, Gilbertsville, PA, USA), and visualized in Odyssey Infrared Imaging System (LI-COR Biosciences, Lincoln, NE, USA).

### 3.7. Chromatin Immunoprecipitation Assay

Chromatin immunoprecipitation was performed with a ChIP assay kit by using antibodies against AcH3 and H4 or control IgG according to the manufacturer’s protocol. The purified DNA was used as template for qPCR amplification using p21 promoter specific primer [32].

### 3.8. Statistical Analyses

Results are expressed as means ± standard deviation of at least two independent experiments, each conducted in triplicate. Differences between control and SVP treatment were analyzed by one-way ANOVA. Differences were considered significant at *p* < 0.05.

## 4. Conclusions

In summary, the present study demonstrated that low dose (0.2 to 0.5 mM) of SVP may induce cell senescence in human hepatocarcinoma cells, which may correlate with the hyperacetylation of histone H3 and H4, up-regulation of p21, and inhibition of RB phosphorylation. Since these doses are clinical available, whether clinical dose of SVP may induce cell senescence in clinical hepatocarcinoma is worthy of further study.
